# Low-Coordinate Mixed Ligand NacNac Complexes of Rare Earth Metals

**DOI:** 10.3390/molecules28041994

**Published:** 2023-02-20

**Authors:** Svetlana V. Klementyeva, Taisiya S. Sukhikh, Pavel A. Abramov, Andrey I. Poddel’sky

**Affiliations:** 1 Chemistry Department, University of Tübingen, Auf der Morgenstelle 18, 72076 Tübingen, Germany; 2 Institute of Inorganic Chemistry, Karlsruhe Institute of Technology (KIT), Engesserstraße 15, 76131 Karlsruhe, Germany; 3 Nikolaev Institute of Inorganic Chemistry SB RAS, Akademika Lavrentieva Ave. 3, 630090 Novosibirsk, Russia

**Keywords:** rare earth, mixed-ligand, β-diketiminate, redox-active ligand, synthesis, X-ray

## Abstract

We report the synthesis and characterization of two types of new mixed-ligand rare earth complexes: tetracoordinate (NacNac^Mes^)Ln(BIAN^dipp^) (Ln = Dy (**1**), Er (**2**) and Y (**3**)) and pentacoordinate (NacNac^Mes^)Ln(AP^dipp^)(THF) (Ln = Dy (**4**), Er (**5**) and Y (**6**)). The first three compounds were prepared by the reaction of [(BIAN^Dipp^)LnI] with potassium β-diketiminate. The salt metathesis of β-diketiminato-supported rare earth dichlorides (NacNac^Mes^)LnCl_2_(THF)_2_ with sodium o-amidophenolate results in compounds **4**–**6**. The crystal structures of complexes **1**–**6** were determined by single-crystal analysis. The combination of bulky monoanionic N-mesityl-substituted β-diketiminates with sterically hindered redox-active ligands led to the very low coordination numbers of rare earths and strong distortion of the chelate ligands.

## 1. Introduction

The combination of spectator ligands with different types of redox-active ligands constitutes a growing area of research in the coordination chemistry of rare earths over the past decades. The bulky N-aryl-substituted β-diketiminates or “NacNac” ligands with a general formula {ArNC(R)CHC(R)NAr}^−^ are one of the most widely used ancillary ligands of post-metallocene series used for stabilizing rare earth complexes [1,2]. They readily form strong Ln-N (Ln = rare earth metal) bonds within six-membered chelating rings with bonding modes ranging from purely σ to a combination of σ and π donation. The easy-tunability of steric and electronic properties which can be implemented by an appropriate choice of starting β-diketones and anilines complete the utility of such ligands. Besides the classical role of spectator ligands, β-diketiminates behave like non-innocent ligands and may be involved in different transformations including redox reactions and metal-ligand cooperative activation of substrates [3].

The development of coordination chemistry of redox-active ligands with rare earths is of undoubted interest from the point of view of both fundamental science and applied research. The combination of a rare earth element with organic ligands which have extended redox properties and several reduction states will make it possible to obtain compounds possessing a number of useful chemical properties [4,5,6,7,8,9,10] as well as properties essential for the development of new magnetic materials [11,12,13,14]. In general, among such properties are the presence of different paramagnetic centers, several redox transitions, anisotropy of magnetic properties at the rare earth metal center, and modulating the magnetic behavior due to both the metal center and the organic redox-active ligand.

The most distinctive feature of redox active ligands (e.g., o-iminoquinones and alpha-diimines) is the possibility of their redox transformations in the metal coordination sphere, which extends their reactivity to a great extent [15,16,17,18,19,20,21,22]. For a long time, o-iminoquinonato ligands have been used in the coordination chemistry of transition metals and a wide range of complexes have been synthesized to date [23,24,25]. As for main-group metals, a fair number of them are also known [26,27,28]. Notably, the combination “redox-active ligand—main group element” allows for the modeling of the reaction abilities of transition metals. For example, it has been shown that antimony(V) o-amidophenolates bind molecular oxygen in a reversible manner in mild conditions [29,30,31,32]. To the best of our knowledge, it is the first example of the main group metal complexes involved in reversible oxygen fixation. BIAN^dipp^, 1,2-bis[(2,6-diisopropylphenyl)imino]acenaphthene, is a redox-active diimine ligand possessing the conformational rigidity of the diimine moiety and pronounced steric hindrances around nitrogen atoms. The unique reactivity of the main-group metal complexes with BIAN^dipp^ ligand was shown by Fedushkin et al. in the examples of addition (in some cases, reversible) and activation of substituted alkynes, alkenes [33,34,35,36,37,38]. It would be very interesting to combine sterically hindered NacNac ligands with redox-active sterically hindered o-iminobenzoquinones and bis-iminoacenaphthenes in a coordination sphere of rare earth.

Huge potential for obtaining mixed-ligand complexes is hidden in the dihalide complexes with a common formula [(NacNac)LnX_2_] (X = Cl [39,40,41,42,43,44,45,46,47], I [48]) via further metathesis of Ln-halide bonds. For example, [(NacNac^Mes^)YCl_2_(THF)_2_] supported by N-mesityl substituted β-diketiminato ligand (NacNac^Mes^ = 2,4,6-Me_3_C_6_H_2_-NC(Me)CHC(Me)N-2,4,6-Me_3_C_6_H_2_) was used for the synthesis of mixed-ligand complexes β-diketiminato catecholate derivatives [49]. The salt metathesis reactions of [(NacNac^Mes^)ErCl_2_(THF)_2_] with potassium salt of doubly reduced diazabutadiene (DAD^Mes^)K_2_ (DAD^Mes^ = 2,4,6-Me_3_C_6_H_2_-NC(Me)-C(Me)N-2,4,6-Me_3_C_6_H_2_) prepared in situ, afforded a new mixed-ligand erbium complex [LEr(CH_2_-DAD^Mes^)(THF)] containing dianionic DAD ligand with an unusual double bond and hydrogen redistribution (CH_2_-DAD^Mes^ = 2,4,6-Me_3_C_6_H_2_-NC(H)(Me)-C(=CH_2_)N-2,4,6-Me_3_C_6_H_2_) [50]. Recently, a series of 2,6-diisopropylphenyl-substituted β-diketiminato-diiodides [(NacNac^dipp^)LnI_2_(THF)_n_] (NacNac^dipp^ = 2,6-^i^Pr_2_C_6_H_3_-NC(Me)CHC(Me)N-2,6-^i^Pr_2_C_6_H_3_); Ln = Dy, Gd, Tb [51], Nd, Sm [52]) was involved in the salt metathesis reactions with potassium phenyl- and 2-pyridylthiolates resulting in β-diketiminato-thiolato complexes. A comprehensive study of divalent samarium β-diketiminato-supported iodides [(NacNac^dipp^)SmI(THF)_n_] revealed a series of polynuclear compounds with different structures depending on the THF content [53].

Here we report two types of mixed-ligand rare earth complexes with spectator monoanionic β-diketiminate ligands and sterically hindered dianionic diamides or o-amidophenolates. Two different synthetic routes were elaborated which are essentially based on the salt metathesis reactions. The first one includes a direct reduction of BIAN^dipp^ with metallic rare earth excess in the presence of iodine to obtain in situ rare earth precursors [(BIAN^dipp^)LnI] (Ln = Dy, Er, Y), allowing further reaction with potassium salt of β-diketiminate (NacNac^Mes^)K affording four-coordinate complexes [(NacNac^Mes^)Ln(BIAN^dipp^)] (**1**–**3**). In contrast to this, five-coordinate complexes [(NacNac^Mes^)Ln(AP^dipp^)(THF)] (**4**–**6**) were obtained by the salt metathesis of disodium o-amodophenolate with β-diketiminate rare earth precursors [(NacNac^Mes^)LnCl_2_]. Compounds **1**–**6** were characterized by standard analytic methods including single-crystal X-ray diffraction analysis which reveals unusually low coordination numbers of rare earths.

## 2. Results and Discussion

### 2.1. Synthesis and Characterization

**Scheme 1:**
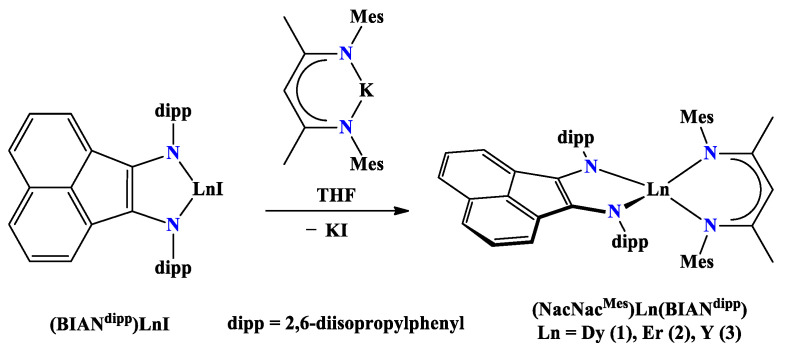
Synthesis of complexes (NacNac^Mes^)Ln(BIAN^dipp^) **1**–**3**.

Heteroligand erbium(III), dysprosium(III), and yttrium(III) NacNac-BIAN complexes **1**–**3** were prepared by the metathesis reaction from the corresponding (BIAN^dipp^)LnI precursors with potassium salt (NacNac^Mes^)K (Scheme 1). Complexes represent a rare type of true tetrahedral heteroligand lanthanide complexes with redox-active ligands. To the best of our knowledge, the tetracoordinate BIAN lanthanide complexes are represented only by homoligand ionic La(III) and Yb(III) derivatives [(BIAN^dipp^)_2_Ln^III^]^+^[(BIAN^dipp^)Ln(DME)_2_]^−^, and neutral Sm(III) complex (BIAN^dipp^)_2_Sm [54]. However, there are no corresponding tetracoordinate lanthanide heteroleptic NacNac-BIAN complexes in the literature to date. A few examples of low-coordinate lanthanide complexes are represented by extremely sterically shielded dipp-1,4-disubstituted diazabutadiene (DAD^dipp^) derivatives of Sm, Yb, Dy, Er [55,56,57].

**Scheme 2:**
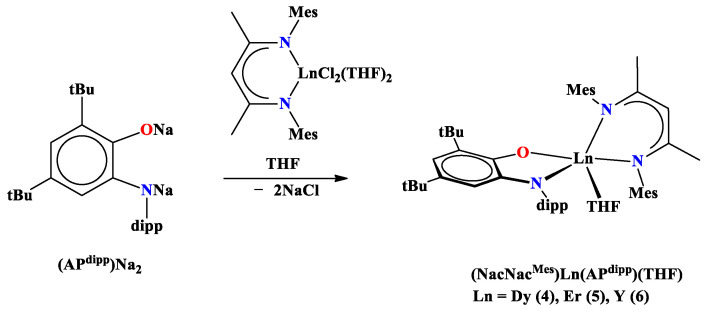
Synthesis of complexes (NacNac^Mes^)Ln(AP^dipp^)(THF) **4**–**6**.

The related erbium(III), dysprosium(III) and yttrium(III) NacNac-AP complexes **4**–**6** have been synthesized by the metathesis reaction between lanthanide β-diketoiminate dichloride (NacNac^Mes^)LnCl_2_(THF)_2_ with sodium o-amidophenolate (AP^dipp^)Na_2_ (Scheme 2).

We have to note that the same metathesis reaction of lanthanide dichloride complexes supported by dipp-substituted diketoiminate (NacNac^dipp^)LnCl_2_(THF)_2_ with sodium 3,5-di-tert-butyl-catecholate (3,5-Cat)Na_2_ in THF afforded dimeric mixed-ligand complexes {(NacNac^dipp^)Ln(m-3,5-Cat)}_2_ with two bridging catecholate ligands (via one m-oxygen atom of each catecholate) between two lanthanide atoms [49]. Thus, the increase of steric shielding with o-amidophenolate ligand as compared with 3,5-di-tert-butyl-catecholate results in the formation of mononuclear derivatives **4**–**6** with a coordinated THF molecule. The five-coordinate mononuclear rare earth o-amidophenolates were not known. There were only binuclear rare earth complexes of these ligands with coordination number 5, or mononuclear complexes with coordination numbers 6 to 8, represented in the literature.

Complexes were found to be extremely sensitive to the traces of air oxygen and moisture. The compositions of compounds **1**–**6** were confirmed by elemental analysis and IR spectroscopy (Figures S1–S6), and NMR spectroscopy (for yttrium complexes **3**, **6**) (Figures S7–S9). The strong absorptions in the range of 1550–1570 cm^−1^ in the IR spectra confirm the partial C=N double-bond character of β-diketiminato NacNac ligand in complexes [39,40,41,42,43,44,45,46,47,48,58]; the well-distinguishable absorptions at 1190–1260 cm^−1^ are due to the valence vibrations of ordinary C–N or C–O bonds typical for doubly reduced forms of redox-active ligands (dianion BIAN in complexes **1**–**3** and dianion AP in complexes **4**–**6**, respectively). In addition, the medium absorption bands near 1640–1650 cm^−1^ correspond to the stretch vibrations of conjugated C=C double bonds in the doubly reduced BIAN ligand (in **1**–**3**) [33,34,35,36,37,38].

### 2.2. X-ray Structures

**Figure 1:**
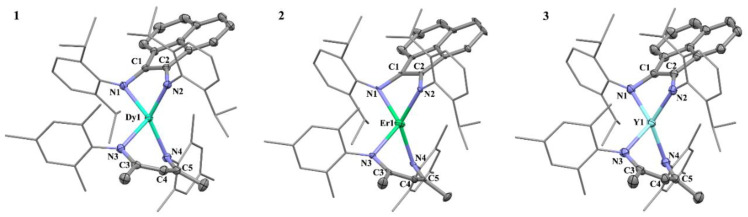
The molecular structures of dysprosium (**1**), erbium (**2**), and yttrium (**3**) complexes of the type (NacNac^Mes^)Ln (BIAN^dipp^). The ellipsoids of 50% probability. The hydrogen atoms are hidden.

**Figure 2:**
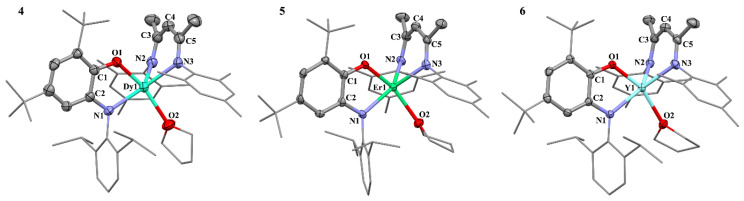
The molecular structures of dysprosium (**4**), erbium (**5**), and yttrium (**6**) complexes of the type (NacNac^Mes^)Ln(AP^dipp^)(THF). The ellipsoids of 50% probability. The hydrogen atoms are hidden.

The molecular structures of complexes **1**–**6** in crystal state were determined by single crystal X-ray diffraction analysis (Figure 1 and Figure 2, Figures S10–S15 of ESI). The dysprosium complex **1** crystallizes from n-hexane solution in the monoclinic space group *P* 2_1_/n, while the isostructural erbium **2** and yttrium **3** compounds crystallize from toluene in the triclinic space group *P*-1, and the unit cells contain one molecule of toluene per complex molecule. All complexes have a monomeric mononuclear structure in the solid state. The small differences in the structures of complexes are concerned with a weak variety of ligand conformations, distances, and angle values. The molecular structures of complexes **1**–**3** are shown in Figure 1 with the selected bond lengths and angles given in Table 1.

The coordination sphere of rare earth atoms in complexes **1**–**3** consists of two bidentate chelating ligands—monoanionic β-diketiminate and dianionic BIAN^dipp^ providing a coordination number of four. The Continuous Symmetry Measures (CSM) analysis [59,60] gave the description of the polyhedron as a tetrahedron as the best fit (Table S2). The index *t*_4_ proposed by L. Yang, D.R. Powell, and R.P. Houser [61] for the description of four-coordinate geometry was calculated to be 0.68 for complex **1**, 0.69 for complex **2**, and 0.72 for complex **3** (*t*_4_ = 1.00 for a perfect tetrahedral geometry, and *t*_4_ = 0.00 for a perfect square planar geometry).

The angles between planes Ln1N1N2 and Ln1N3N4 are 82.42°, 89.26°, and 89.18° in complexes **1**–**3**, respectively. Both N,N′-ligands are asymmetrically coordinated to the rare earth metals with the acute N-Ln-N angles and slight differences of Ln1–N1/Ln1–N2 and Ln1–N3/Ln1–N4 bond lengths (Table 1), which are well comparable with other lanthanide compounds bearing such ligands [39,40,41,42,43,44,45,46,47,48,49]. The bond distances in the N3C3C4C5N4 backbone confirm the delocalization within the π-system of monoanionic β-diketiminato ligands. The bond lengths in BIAN^dipp^ indicate unambiguously a dianionic state of the ligand [33,34,35,36,37,38] with the characteristic N–C bond lengths lying in the range of 1.399–1.409 Å for complexes **1**–**3** (Table 1). The chelate rings are strongly bent almost along the nitrogen-nitrogen lines: The angles between Ln1N1N2 and N1C1C2N2planes in the fragment Ln(BIAN^dipp^) in complexes **1**, **2**, and **3** are 50.49, 49.95, and 47.13°, respectively. Between Ln1N3N4 and N3C3C5N4 planes in the fragment Ln(NacNac^Mes^), the angles are 59.76, 60.81, and 56.11°, respectively. Such distortion of the chelate rings in complexes **1**–**3** is one of the biggest in the BIAN^dipp^ or NacNac^Mes^ lanthanide complexes that is caused by the large degree of steric saturation with bulky BIAN^dipp^ or NacNac^Mes^ ligands. The closest values for such a distortion of the BIAN^dipp^ chelate ring were found in very sterically shielded bis-BIAN^dipp^ complexes of tetracoordinate Yb(III), La(III), Sm(III) [54], where this bent angle is in the range of 50–55°, or in heptacoordinate (BIAN^dipp^)La(DME)_2_I (46.8°) as well as in mixed-ligand [(BIAN^dipp^)LaCp*I]^−^ and (BIAN^dipp^)LaCp*(THF) (48.2 and 51.5°) [62] or (BIAN^dipp^)SmCp*(THF) (47.54°) [63]. In addition, to minimize steric repulsion between the bulky ligands, the N-aryl rings lie close to perpendicular to the NCCN planes of both ligands, and rare earth metal atoms occupy the positions out of these planes. The deviation of rare earth metals from the corresponding plane N1C1C2N2 of BIAN^dipp^ is equal to 1.26(1), 1.32(1), and 1.21(1) Å, while the deviation from the N3C3C5N4 plane of β-diketiminato ligand amounts to 1.51(1), 1.62(1), and 1.45(1) Å for complexes **1**–**3**, respectively. The observed deviation is even more pronounced than in the mixed-ligand erbium complex with NacNac^Mes^ and DAD ligands [50]. Due to such deviation, short contacts between the central metal and carbons C1 and C2 of BIAN ligand are observed in all complexes (**1**–**3**): the distances Ln1–C1 and Ln1–C2 are 2.640(2) and 2.636(2) Å for complex **1**, 2.708(3) and 2.741(3) Å for complex **2**, and 2.672(2) and 2.696(2) Å for complex **3**. The same short contacts were found by A.H. Cowley et al. in (BIAN^dipp^)SmCp*(THF) [63].

In contrast to **1**–**3**, the coordination environment of lanthanides in o-iminoquinonato complexes **4**–**6** includes NacNac anionic ligand, dianion o-amidophenolate, and a coordinated THF molecule. According to the CSM analysis, the coordination polyhedron of **4**–**6** can be described either as a spherical square pyramid or a trigonal bipyramid (Table S3). In the former case, two N atoms and two O atoms lie in the square basal plane (Figure S16a), while in the latter, three N atoms lie in the trigonal basal plane (Figure S16b). For compound **4**·Hexane, one can also reasonably describe the polyhedron as a vacant octahedron. The molecular structures of complexes **4**–**6** are shown in Figure 2. The selected bond lengths and angles are given in Table 2.

The redox-active O,N-coordinated ligand is obviously in a dianionic state, which is confirmed by the single O–C (1.343–1.352 Å) and N–C (1.386–1.404 Å) bonds typical for different o-amidophenolate complexes (1.34–1.36 and 1.38–1.41 Å, respectively) [30,31,32,63,64,65,66,67], as well as by aromatic C–C bonds in six-membered carbon cycles (with the average distances of 1.400, 1.406 and 1.401 Å for **4**–**6**, respectively). The NacNac ligands in **4**–**6** have the same structural features as those in **1**–**3**. However, the metallocycles Ln1N2C3C4C5N3 in **4**–**6** are not bent as dramatically as in **1**–**3**: the angles between the Ln1N1N3 and N2C2C4N3 planes in the fragments Ln(NacNac^Mes^) are equal to 34.45°, 36.17° and 36.07°, respectively (in contrast to 59.76, 60.81, and 56.11° in **1**–**3**). At the same time, the chelate metallocycles Ln1O1C1C2N1 in **4**–**6** are very close to planar (the corresponding bent angles along O1···N1 line are 2.45°, 1.16° and 1.00° only). This observation confirms the decrease of steric saturation caused by NacNac^Mes^, AP^dipp^ and THF in **4**–**6** as compared to the steric hindrances from NacNac^Mes^ and BIAN^dipp^ in **1**–**3**. The deviations of rare earth metals from the corresponding plane O1C1C2N2 of AP^dipp^ in **4**–**6** are very small (0.076, 0.036, and 0.030 Å), and the deviation of Ln1 from the N2C3C5N3 plane of β-diketiminato ligand are 1.005(1), 1.015(1) and 1.026(1) Å, only.

In the unit cells, molecules of BIAN complexes **1**–**3** (Figures S10–S12), as well as AP complexes **4**–**6** (Figures S13–S15), are isolated with only the usual intermolecular van der Waals interactions observed in crystal. Therefore, no dimeric particles are observed in the crystal state.

## 3. Materials and Methods

### 3.1. General

All manipulations of air- and moisture-sensitive materials were performed with the rigorous exclusion of oxygen and moisture in flame-dried Schlenk-type glassware either on a dual-manifold Schlenk-line, interfaced to a high vacuum (10^−3^ mbar) line, or in an argon-filled MBraun or Korea Kiyon KK-021AS glovebox. THF and toluene were distilled under nitrogen from potassium benzophenone ketyl prior to use. *n*-Hexane was distilled under nitrogen over Na/K alloy prior to use. All solvents for vacuum line manipulations were stored in vacuo over Na/K alloy in resealable flasks. IR spectra were obtained in KBr pellet by means of a FT-801 Fourier spectrometer (Simex, Saint Petersburg, Russia) (complexes **1**–**3**) and a Bruker FTIR Tensor 37 instrument by the attenuated total reflection method (ATR) (complexes **4**–**6**) (Billerica, MA, USA). NMR spectra for **3** and **6** were recorded with a Bruker Avance 500 in benzene-d_6_. Chemical shifts were referenced to internal solvent resonances and were reported relative to tetramethylsilane (^1^H and ^13^C{^1^H} NMR spectroscopy). Elemental analysis (C,H,N) was carried out with a Euro EA 3000 analyzer (Eurovector, Pavia, Italy). Starting materials [(NacNac^Mes^)LnCl_2_(THF)_2_] [1], (NacNac^Mes^)K [40], [BianLnI] [62], IQ [68] and Bian^dipp^ [69] were prepared according to literature procedures.

### 3.2. Synthesis and Characterization

#### 3.2.1. Synthesis of (NacNac^Mes^)Ln(Bian^dipp^) (Complexes **1**–**3**)

THF (15 mL) was condensed onto a mixture of BIAN^dipp^ (100 mg, 0.2 mmol), iodine (26 mg, 0.1 mmol), and an excess of metallic rare earth (810 mg Dy; 840 mg Er; 450 mg Y, 5 mmol) with a continuous stirring. The mixture was stirred for 72 h at room temperature in order to reach an exhaustive reduction of BIAN^dipp^ while the color was turning dark blue. The obtained dark-blue solution was added with stirring to a solution of (NacNac^Mes^)K (75 mg, 0.2 mmol) in THF (10 mL) resulting in the dark-blue solution and the gradual precipitation of KI. The mixture was stirred for 24 h at room temperature and then KI was filtered off. THF was evaporated under reduced pressure to dryness and the residue was dissolved in toluene (15 mL). The toluene solution was stirred at 90 °C for 24 h with the subsequent evaporation of the toluene to dryness. *n*-Hexane (10 mL) was condensed on the dark-blue residue and allowed to stay for a couple of days. In the case of (NacNac^Mes^)Dy(Bian^dipp^) (**1**), dark-blue cubic crystals of **1** (103 mg, yield 52%) suitable for single-crystal X-ray diffraction were grown from this solution. In the case of (NacNac^Mes^)Er(Bian^dipp^) (**2**) and (NacNac^Mes^)Y(Bian^dipp^) (**3**), no crystals were obtained from the *n*-hexane solutions. Thus, *n*-hexane was evaporated to dryness and the residues were redissolved in toluene (10 mL). The toluene solutions were concentrated to approximately half their volume and allowed to stay for two weeks, affording dark-blue block-shaped crystals of **2**·C_7_H_8_ (61 mg, yield 28%) and **3**·C_7_H_8_ (55 mg, yield 27%) suitable for single-crystal X-ray diffraction.

**1**: Anal. calcd (%) for C_59_H_69_N_4_Dy (996.75): C 71.10, H 6.98, N 5.62; found C 70.74, H 6.91, N 5.47%. IR ν, cm^−1^: 568(w), 624(w), 715(w), 755(m), 768(m), 797(m), 817(m), 855(m), 919(w), 933(w), 956(w), 1013(m), 1035(m), 1058(w), 1103(w), 1143(m), 1193(m), 1223(w), 1256(m), 1307(m), 1331(m), 1362(s), 1380(s), 1395(s), 1441(s), 1456(s), 1478(s), 1527(s), 1552(s), 1570(s), 1622(m), 2866(s), 2919(s), 2961(s).

**2**·C_7_H_8_: Anal. calcd (%) for C_66_H_77_N_4_Er (1093.60): C 72.49, H 7.10, N 5.12; found C 72.14, H 6.95, N 4.98%. IR ν, cm^−1^: 564(w), 624(w), 695(w), 728(m), 755(m), 767(m), 796(m), 816(m), 853(m), 919(w), 933(w), 956(w), 1011(m), 1035(m), 1058(w), 1108(w), 1143(m), 1191(m), 1223(w), 1256(m), 1305(m), 1331(m), 1362(s), 1382(s), 1395(s), 1443(s), 1457(s), 1476(s), 1522(m), 1554(s), 1570(s), 1624(m), 2866(s), 2919(s), 2961(s).

**3**·C_7_H_8_: Anal. calcd (%) for C_66_H_77_N_4_Y (1015.25): C 78.08, H 7.65, N 5.52; found C 77.51, H 7.38, N 5.34%. IR ν, cm^−1^: 564(w), 625(w), 695(w), 728(w), 755(m), 767(m), 797(m), 817(m), 854(m), 915(w), 934(w), 958(w), 1013(m), 1035(m), 1058(w), 1107(w), 1143(m), 1193(m), 1221(w), 1256(m), 1305(m), 1331(m), 1362(s), 1382(s), 1395(s), 1442(s), 1456(s), 1478(s), 1520(m), 1554(s), 1570(s), 1624(m), 2866(s), 2919(s), 2961(s). ^1^H NMR (500 MHz, C_6_D_6_, δ, ppm): 0.68 (br. s., 3H, CH_3_ of Mes), 0.95–1.15 (br. m., 12H, 4CH_3_ of iPr), 1.26–1.55 (br. m., 9H, 3CH_3_ of iPr), 1.56–1.68 (br. m., 9H, 2CH_3_ of Mes and 1CH_3_ of iPr), 1.90 (s., 6H, 2CH_3_ of NacNac), 2.00 (s., 6H, 2CH_3_ of Mes), 2.33 (br.s., 3H, CH_3_ of Mes), 2.87 (br.m., 1H, CH of iPr), 3.65–3.80 (br. m., 3H, 3CH of iPr), 5.62 (s, 1H, CH of NacNac), 6.02 (br.s., 1H, arom.), 6.17 (br.d., ^3^J(H,H) = 6.4 Hz, 1H, arom.), 6.42 (br.d., ^3^J(H,H) = 6.4 Hz, 1H, arom.), 6.50–6.65 (br. m., 3H, arom.), 6.80–6.85 (br.s., 1H, arom.), 6.95–7.07 (m., 4H, arom.), 7.10–7.17 (m., 2H, arom.), 7.25–7.37 (br.m., 3H, arom.).

#### 3.2.2. Synthesis of (NacNac^Mes^)Ln(AP^dipp^) (**4**–**6**)

A solution of (AP^dipp^)Na_2_ in THF (15 mL) (obtained by the exhaustive reduction of IQ (114 mg, 0.3 mmol) with the sodium excess) was added to a solution of [(NacNac^Mes^)LnCl_2_(THF)_2_] (213 mg Dy; 215 mg Er; 191 Y, 0.5 mmol) in THF (20 mL) resulting in the orange solution and the gradual precipitation of NaCl. The mixture was stirred for 48 h at room temperature and then NaCl was removed by filtration. Then THF was evaporated to dryness, and the orange residue was treated with two portions of *n*-hexane (2 × 10 mL) and subsequent evaporation to afford an orange solid. The solid was extracted with *n*-hexane in the two-section sealed ampoule. The concentration of the orange *n*-hexane extract by slow evaporation for two days afforded orange crystals of **4** 0.5Hexane (185 mg, yield 62%), **5** (145 mg, yield 51%) and **6** (85 mg, yield 32%) suitable for single-crystal X-ray diffraction.

**4**·0.5Hexane: Anal. calcd (%) for C_56_H_81_N_3_O_2_Dy (990.76): C 67.89, H 8.24, N 4.24; found C 67.73, H 8.11, N 4.27%. IR ν, cm^−1^: 567(w), 625(w), 652(w), 698(w), 762(w), 797(w), 854(s), 916(w), 976(w), 1015(m), 1144(m), 1192(s), 1219(w), 1250(s), 1310(w), 1327(m), 1379(s), 1439(s), 1466(s), 1514(s), 1551(s), 1624(w), 1659(w), 2866(s), 2918 (s), 2959(s).

**5**: Anal. calcd (%) for C_53_H_74_N_3_O_2_Er (952.43): C 66.84, H 7.83, N 4.41; found C 66.64, H 7.91, N 4.47%. IR ν, cm^−1^: 569(w), 629(w), 650(w), 704(w), 766(w), 797(w), 854(s), 916(w), 978(w), 1020(m), 11,442(m), 1191(s), 1256(s), 1310(w), 1329(w), 1383(s), 1431(s), 1438(s), 1474(s), 1516(w), 1552(s), 1622(m), 1666(w), 2868(s), 2912 (s), 2960(s).

**6**: Anal. calcd (%) for C_53_H_74_N_3_O_2_Y (874.08): C 72.83, H 8.53, N 4.81; found C 72.69, H 8.37, N 4.71%. IR ν, cm^−1^: 534(w), 651(w), 698(w), 760(w), 794(w), 852(s), 916(w), 979(w), 1019(m), 1142(m), 1190(s), 1255(s), 1327(w), 1382(s), 1439(s), 1472(s), 1518(w), 1553(s), 1624(m), 1662(w), 2865(s), 2919 (s), 2956(s). ^1^H NMR (500 MHz, C_6_D_6_, δ, ppm): 0.76 (br. s., 3H, 1CH_3_ of Mes), 0.98 (br.d., ^3^J(H,H) = 6.2 Hz, 6H, 2CH_3_ of iPr), 1.33 (br.d., ^3^J(H,H) = 6.5 Hz, 6H, 2CH_3_ of iPr), 1.42 (s., 9H, tBu), 1.68 (s., 6H, 2CH_3_ of NacNac), 1.89 (br.s., 9H, tBu), 1.80–1.90 (br.m., 4H, THF), 2.09 (s., 6H, 2CH_3_ of Mes), 2.60 (br.s., 6H, 2CH_3_ of Mes), 2.81 (br. m., 4H, THF), 3.29 (br.m., 2H, 2CH of iPr), 5.27 (s., 1H, CH of NacNac), 6.01 (d., ^4^J(H,H) = 2.3 Hz, 1H, arom. C_6_H_4_), 6.60–6.88 (m., 4H, arom. Mes), 6.90 (d., ^4^J(H,H) = 2.3 Hz, 1H, arom. C_6_H_4_), 7.04 (t., ^3^J(H,H) = 7.5 Hz, 1H, arom. C_6_H_3_), 7.14 (d., ^3^J(H,H) = 7.5 Hz, 2H, arom. C_6_H_3_). ^13^C{^1^H} NMR (125 MHz, C_6_D_6_, δ, ppm): 17.93, 19.95, 20.79, 23.66, 24.53, 24.78, 26.04, 29.18, 30.82, 32.60, 34.75, 35.37, 69.62, 97.02, 108.25, 109.22, 122.67, 123.56, 128.35, 129.46, 130.37, 131.21, 134.09, 137.34, 143.50, 145.01, 150.04, 152.27, 153.86, 166.98.

### 3.3. X-ray Diffraction

X-ray suitable crystals of **1**, **2**·Toluene, **3**·Toluene, **4**·0.5Hexane, **5**, and **6** were covered with mineral oil (Aldrich, St. Louis, MO, USA), selected under a microscope, and mounted on the tips of thin glass fibers. The crystals were transferred directly to the cold stream of a Bruker X8 Apex diffractometer (at 150(2) K for **1**, **2**, **3**) or a STOE IPDS 2 diffractometer (at 200(2) K for **4**, 293(2) K for **5** and 150(2) K for **6**). X-ray intensity data were collected using graphite monochromated Mo Kα radiation (λ = 0.71073 Å). The standard technique was used (φ- and ω-scans of 0.5° frames). The crystal structures were solved using the SHELXT [70] and were refined using the SHELXL [71] programs with OLEX2 GUI [72]. Atomic displacement parameters for non-hydrogen atoms were refined anisotropically, with the exception of some disordered solvent molecules which were refined with DFIX, DANG, ISOR and RIGU restraints. Hydrogen atoms of organic ligands were placed in geometrically idealized positions and refined as riding on their parent C atoms.

The main crystallographic data and structure refinement details for all complexes are presented in Table S1. CCDC 2226117 (**1**), 2236420 (**2**·Toluene), 2236421 (**3**·Toluene), 2236417 (**4**·0.5Hexane), 2236419 (**5**), 2236418 (**6**) contain the supplementary crystallographic data. These data can also be obtained free of charge at ccdc.cam.ac.uk/structures/ (accessed on 12 February 2023) from the Cambridge Crystallographic Data Centre.

## 4. Conclusions

We have prepared a series of low coordinate rare earth complexes (NacNac^Mes^)Ln(BIAN^dipp^) (**1**–**3**) and (NacNac^Mes^)Ln(AP^dipp^)(THF) (**4**–**6**) (Ln = Dy, Er, Y) and shown a dependence of coordination environment of rare earth central metals on the bulkiness of the ligands. The salt metathesis reaction starting from either Ln-Cl or Ln-I derivatives proved to be a very prospective route to access the desired mixed-ligand complexes with various mono- and dianionic ligands. The neutral BIAN^Dipp^ is able to be reduced with rare earth metals excess in the presence of iodine and further utilized in the reaction with potassium β-diketiminate, thus affording the unique four-fold coordinated complexes **1**–**3**. The central rare earth atoms are coordinated only with two bidentate N,N-ligands without any solvate molecule. The less sterically hindered o-amidophenolate AP^Dipp^, formally with one oxygen atom instead of N-aryl group, enables a formation of five-fold coordinated complexes **4**–**6** by the reaction of β-diketiminato supported rare earth dichlorides (NacNac^Mes^)LnCl_2_(THF)_2_ with sodium o-amidophenolate. Along with three nitrogen and one oxygen atom of two chelating ligands, the fifth position in the coordination sphere of rare earth atoms in **4**–**6** is occupied by a coordinated THF molecule.

Firstly, it should be noted that such a strong steric hindrance both of monoanionic N-mesityl substituted β-diketiminates and redox-active diimine or iminoquinone ligands leads to very low coordination and, in particular, extremely rare complexes of rare earths with severely distorted tetrahedral (non-solvate) and trigonal-bipyramidal geometry.

Secondly, the presence of two such bulky ligands in the coordination sphere of rare earths leads to the strong distortion of the ligands chelate cycles, which reduces the steric tension in the complexes as a whole.

## Figures and Tables

**Table 1:** The selected bond lengths and angles in complexes **1**–**3** in crystals according to X-ray diffraction.

Complex	1 (Ln = Dy)	2 (Ln = Er)	3 (Ln = Y)
Bond, Å			
Ln1–N1	2.204(2)	2.250(3)	2.184(2)
Ln1–N2	2.208(2)	2.309(3)	2.235(2)
Ln1–N3	2.303(2)	2.385(3)	2.289(2)
Ln1–N4	2.321(2)	2.421(3)	2.334(2)
N1–C1	1.399(2)	1.399(5)	1.409(3)
N2–C2	1.407(2)	1.400(5)	1.401(3)
N3–C3	1.320(2)	1.332(5)	1.340(3)
N4–C5	1.340(2)	1.325(6)	1.343(3)
C1–C2	1.411(2)	1.407(5)	1.400(3)
C3–C4	1.427(2)	1.421(6)	1.403(4)
C4–C5	1.411(2)	1.416(6)	1.413(4)
Angle, °			
N1–Ln1–N2	84.47(5)	81.26(12)	83.65(7)
N3–Ln1–N4	81.67(5)	78.98(11)	82.21(7)
torsion N1–C1–C2–N2	−0.06	1.14	1.71
torsion N3–C3–C5–N4	−7.34	0.32	0.96
<(Ln1N1N2, N1C1C2N2)	50.49	49.95	47.13
<(Ln1N3N4, N3C3C5N4)	59.76	60.81	56.11

**Table 2:** The selected bond lengths and angles in **4**–**6** in crystals according to X-ray diffraction.

Complex	4 (Ln = Dy)	5 (Ln = Er)	6 (Ln = Y)
Bond, Å			
Ln1–N1	2.301(3)	2.279(3)	2.284(2)
Ln1–O1	2.110(3)	2.090(2)	2.091(2)
Ln1–N2	2.329(4)	2.273(3)	2.290(3)
Ln1–N3	2.278(3)	2.274(3)	2.285(3)
Ln1–O2	2.411(4)	2.367(3)	2.383(2)
O1–C1	1.352(5)	1.350(4)	1.343(3)
N1–C2	1.386(6)	1.395(4)	1.404(4)
N2–C3	1.331(6)	1.321(5)	1.324(4)
N3–C5	1.338(6)	1.326(5)	1.332(4)
C1–C2	1.435(6)	1.417(5)	1.432(4)
C3–C4	1.410(7)	1.405(6)	1.411(5)
C4–C5	1.404(8)	1.409(6)	1.401(5)
Angle, °			
N1–Ln1–O1	71.54(12)	72.85(10)	72.57(8)
N2–Ln1–N3	79.09(14)	81.69(11)	80.77(10)
O1–Ln1–O2	162.07(13)	160.06(9)	159.70(8)
torsion O1–C1–C2–N1	0.19	2.23	−2.26
torsion N2–C3–C5–N3	1.87	2.19	−2.39
<(Ln1O1N1, O1C1C2N1)	2.45	1.16	1.00
<(Ln1N2N3, N2C3C5N3)	34.45	36.17	36.07

## Data Availability

The data presented in this study are available in this article.

## Supplementary Materials

The following supporting information can be downloaded at https://www.mdpi.com/article/10.3390/molecules28041994/s1, Figures S1–S6 with IR data; Figures S7–S9 with NMR spectroscopic data; Figures S10–S15 with crystal cells; Table S1 with the main crystallographic data and structure refinement details; Tables S2 and S3 with results of the geometry analysis (SHAPE); and Figure S16 showing the overlay of the coordination polyhedrons and regular spherical square pyramid and trigonal bipyramid according to Continuous Shape Measures routine implemented in SHAPE program.

## Sample Availability

Samples of the compounds are available from the authors.
