# Supplementary material for: Low-Coordinate Mixed Ligand NacNac Complexes of Rare Earth Metals
Source: Molecules. 2023 Feb 20;28(4):1994. doi: 10.3390/molecules28041994 (PMC9965685; doi:10.3390/molecules28041994)
Supplement: Supplementary file 1 [file molecules-28-01994-s001.zip › molecules-2199603-supplementary.pdf]

Low-Coordinate Mixed Ligand NacNac complexes of Rare Earth metals

S. Klementyeva, T. Sukhikh, P. Abramov, A. Poddel'sky

Content:

Figure S1. The IR spectrum of $(NacNac^{Mes})Dy(BIAN^{dipp})$ (**1**) 2

Figure S2. The IR spectrum of $(NacNac^{Mes})Er(BIAN^{dipp})$ (**2**) 2

Figure S3. The IR spectrum of $(NacNac^{Mes})Y(BIAN^{dipp})$ (**3**) 3

Figure S4. The IR spectrum of $(NacNac^{Mes})Dy(AP^{dipp})$ (**4**) 3

Figure S5. The IR spectrum of $(NacNac^{Mes})Er(AP^{dipp})$ (**5**) 4

Figure S6. The IR spectrum of $(NacNac^{Mes})Y(AP^{dipp})$ (**6**) 4

Figure S7. The ^{1}H NMR spectrum of $(NacNac^{Mes})Y(BIAN^{dipp})$ (**3**) (500 MHz, C_6D_6). 5

Figure S8. The ^{1}H NMR spectrum of $(NacNac^{Mes})Y(AP^{dipp})$ (**6**) (500 MHz, C_6D_6). 5

Figure S9. The $^{13}C\{^{1}H\}$ NMR spectrum of $(NacNac^{Mes})Y(AP^{dipp})$ (**6**) (125 MHz, C_6D_6). 6

Figure S10. Crystal cell of $(NacNac^{Mes})Dy(BIAN^{dipp})$ (**1**) along a axis. . . . 7

Figure S11. Crystal cell of $(NacNac^{Mes})Er(BIAN^{dipp})$·Toluene (**2**·Toluene) along a axis. . 8

Figure S12. Crystal cell of $(NacNac^{Mes})Y(BIAN^{dipp})$·Toluene (**3**·Toluene) along a axis. . 9

Figure S13. Crystal cell of $(NacNac^{Mes})Dy(AP^{dipp})(THF)$·0.5Hexane (**4**·0.5Hexane) along a axis. 10

Figure S14. Crystal cell of $(NacNac^{Mes})Er(AP^{dipp})(THF)$ (**5**) along a axis. . . . 11

Figure S15. Crystal cell of $(NacNac^{Mes})Y(AP^{dipp})(THF)$ (**6**) along a axis. . . . 12

Table S1. Crystal data and structure refinement for the compounds. . . . 13

Table S2. Geometry analysis (SHAPE) of the tetracoordinated lanthianide polyhedra in **1**–**3** 14

Table S3. Geometry analysis (SHAPE) of the pentacoordinated lanthianide polyhedra in **4**–**6** 14

Figure S16. Overlay of the coordination polyhedron in complexes **4**–**6** (by the example of **6**) and regular spherical square pyramid (a) and trigonal bipyramid (b) according to Continuous Shape Measures routine implemented in SHAPE program 14

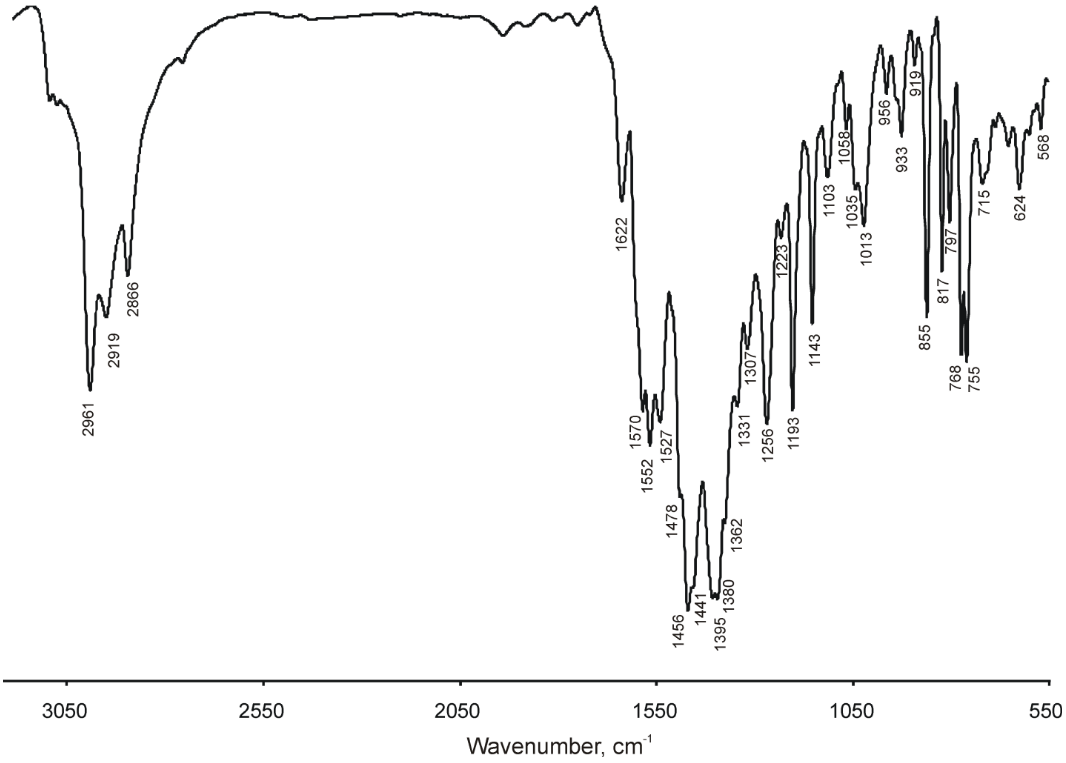

Figure S1. The IR spectrum of (NacNac^{Mes})Dy(BIAN^{dipp}) (**1**) (3200-550 cm^{-1}, KBr).

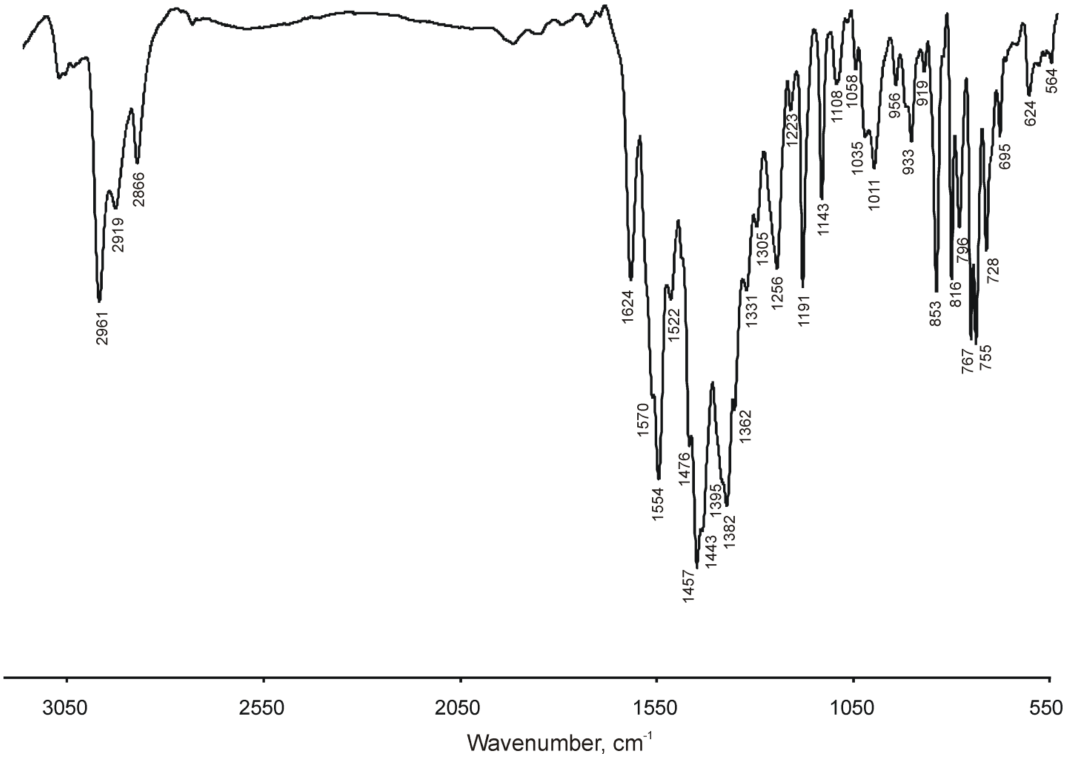

Figure S2. The IR spectrum of (NacNac^{Mes})Er(BIAN^{dipp}) (**2**) (3200-550 cm^{-1}, KBr).

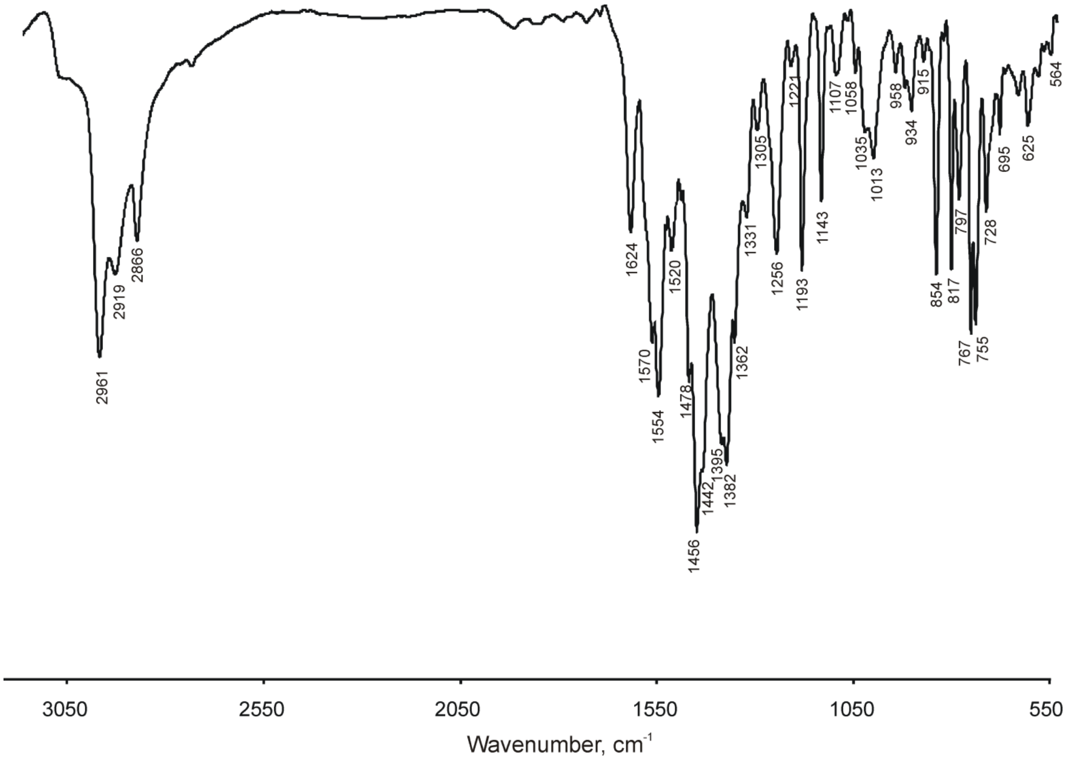

Figure S3. The IR spectrum of (NacNac^{Mes})Y(BIAN^{dipp}) (**3**) (3200-550 cm^{-1}, KBr).

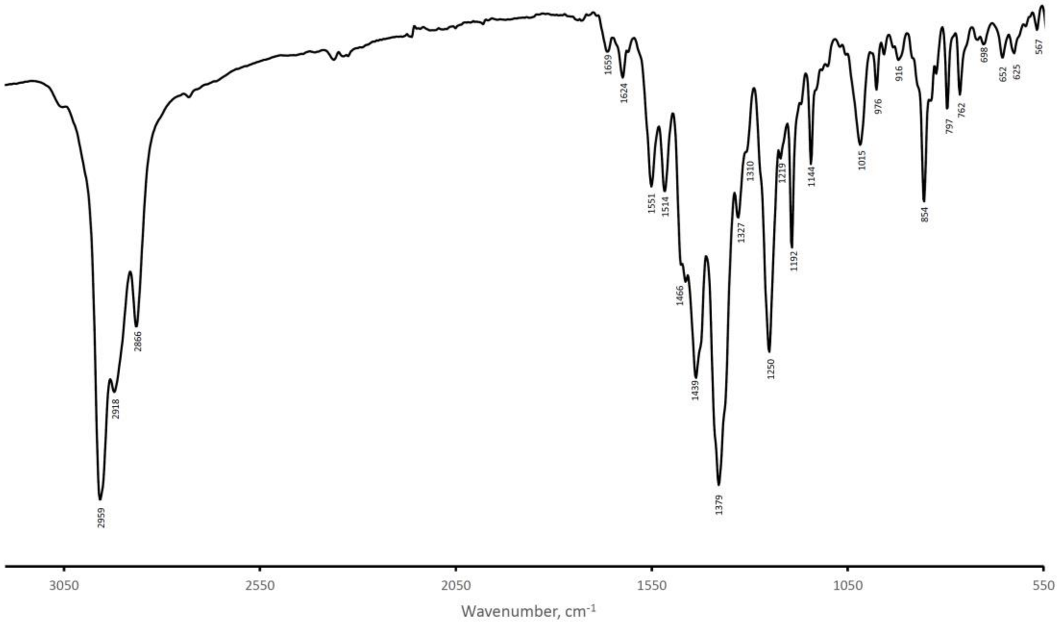

Figure S4. The IR spectrum of (NacNac^{Mes})Dy(AP^{dipp}) (**4**) (3200-550 cm^{-1}, KBr).

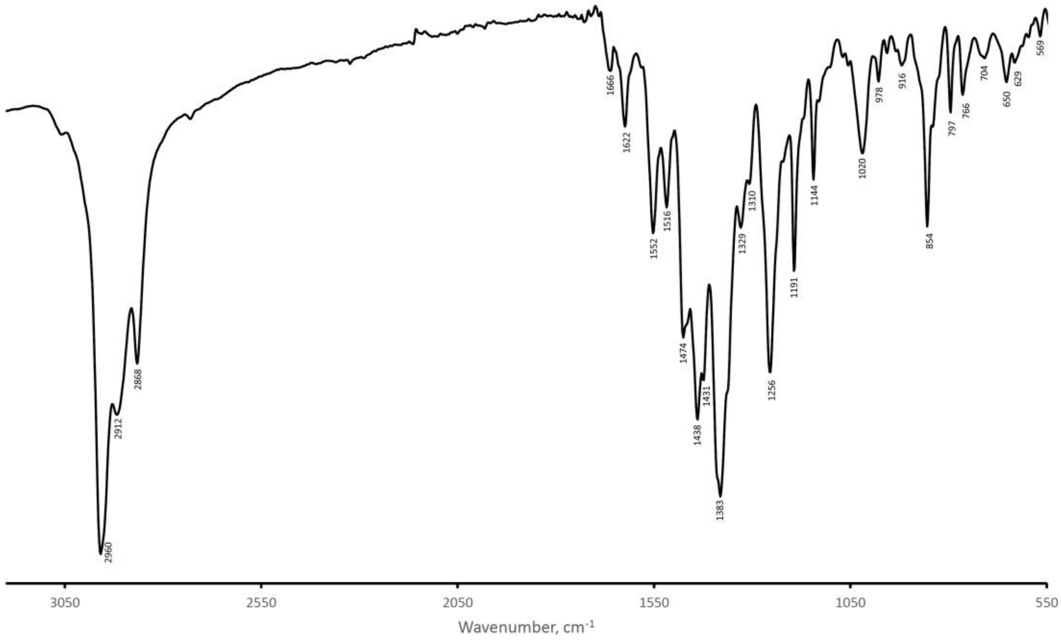

Figure S5. The IR spectrum of (NacNac^{Mes})Er(AP^{dipp}) (**5**) (3200-550 cm^{-1}, KBr).

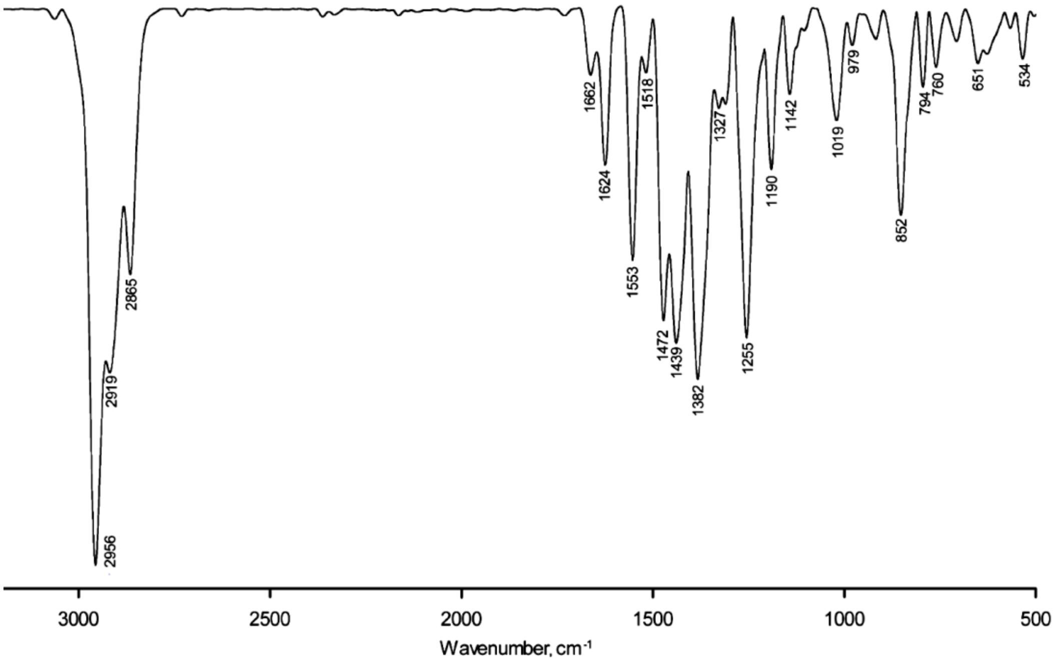

Figure S6. The IR spectrum of (NacNac^{Mes})Y(AP^{dipp}) (**6**) (3200-500 cm^{-1}, KBr).

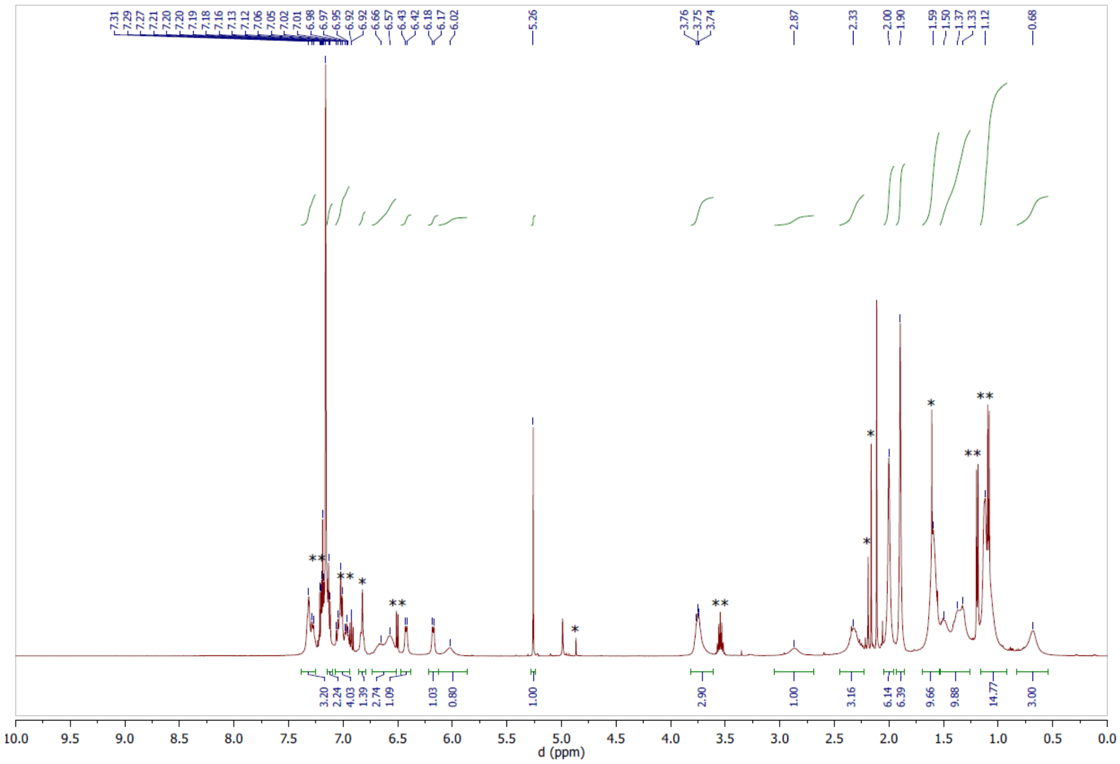

Figure S7. The ^{1}H NMR spectrum of (NacNac^{Mes})Y(BIAN^{dipp}) (**3**) (500 MHz, C_6D_6). * the peaks of NacNac^{Mes}H and ** the peaks of BIAN^{dipp} as impurities due to an extremely high sensitivity of complex to oxygen and moisture traces.

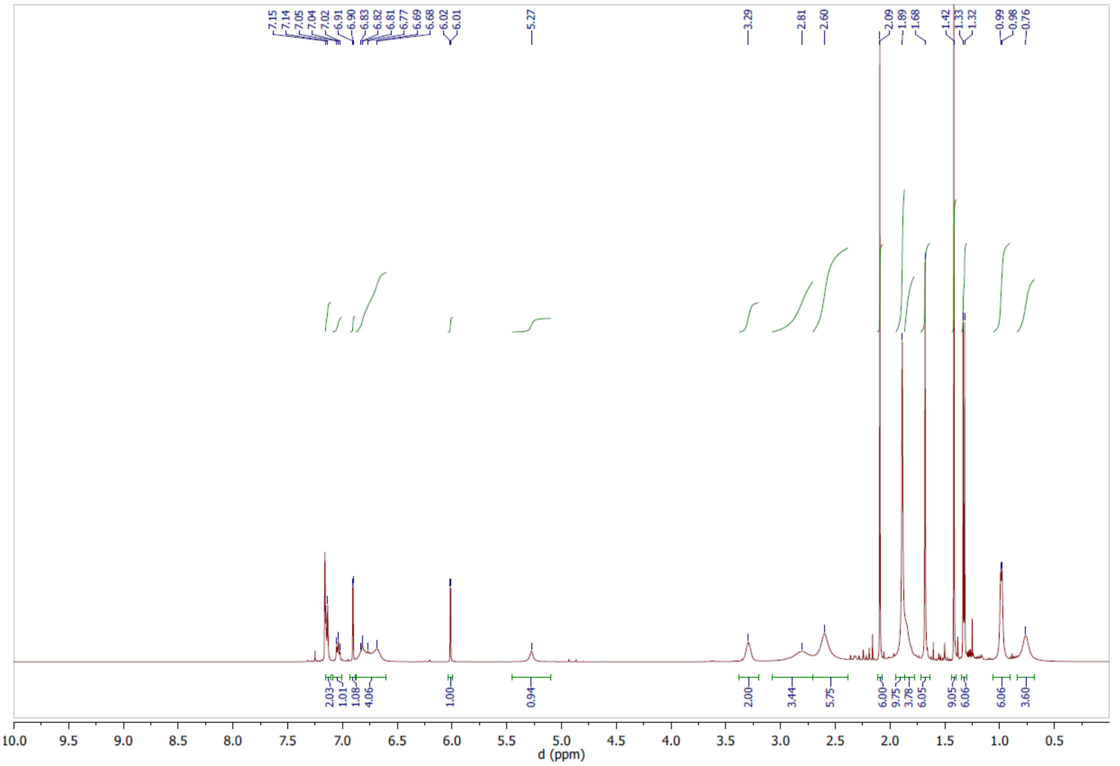

Figure S8. The ^{1}H NMR spectrum of (NacNac^{Mes})Y(AP^{dipp}) (**6**) (500 MHz, C_6D_6).

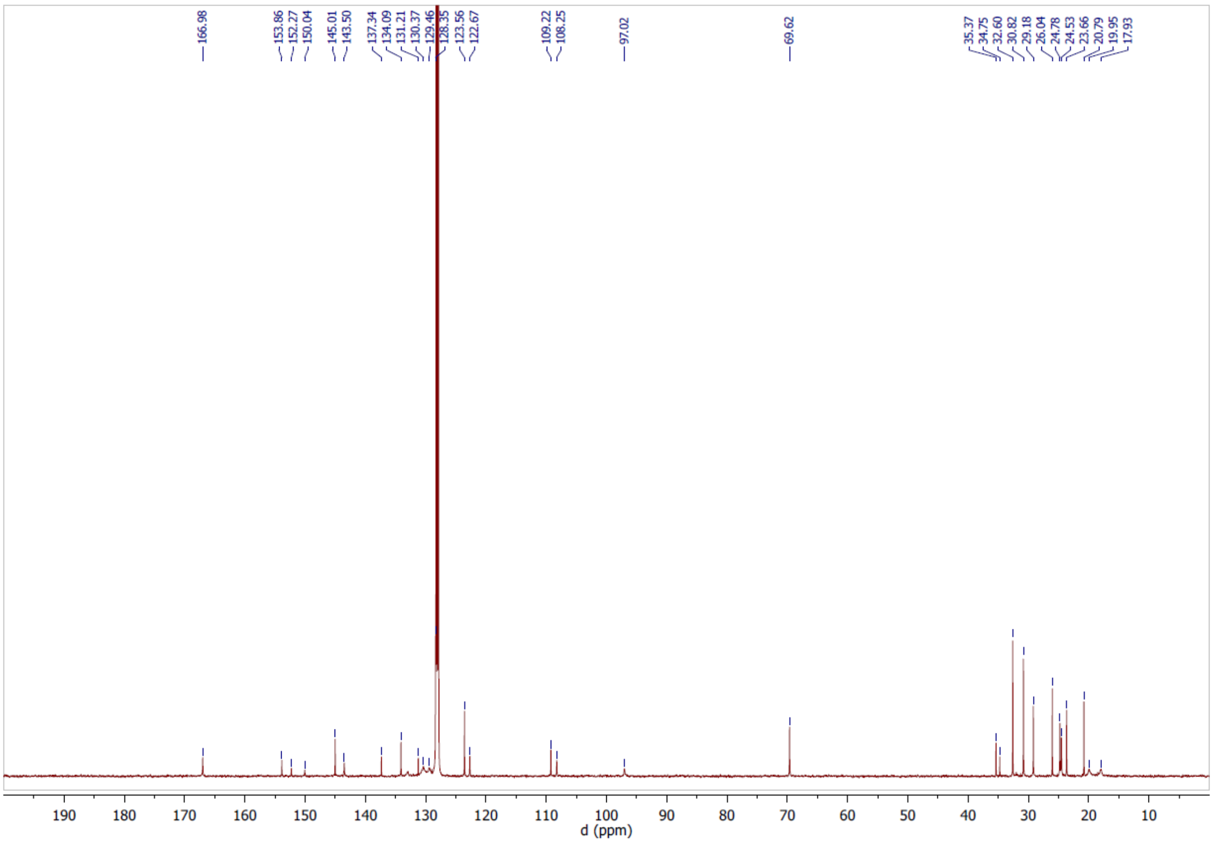

Figure S9. The $^{13}C\{^{1}H\}$ NMR spectrum of $(NacNac^{Mes})Y(AP^{dipp})$ (**6**) (125 MHz, C_6D_6).

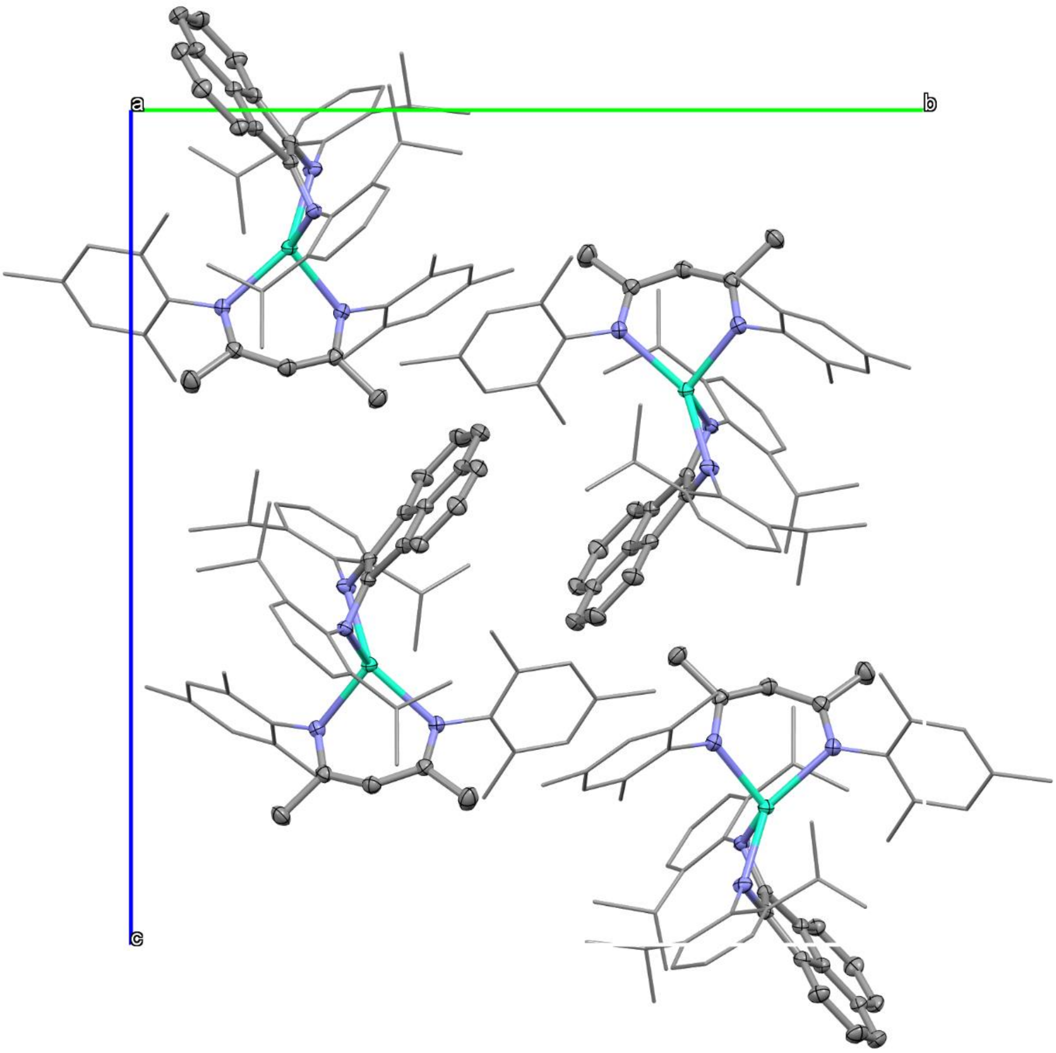

Figure S10. Crystal cell of (NacNac^{Mes})Dy(BIAN^{dipp}) (**1**) along a axis.

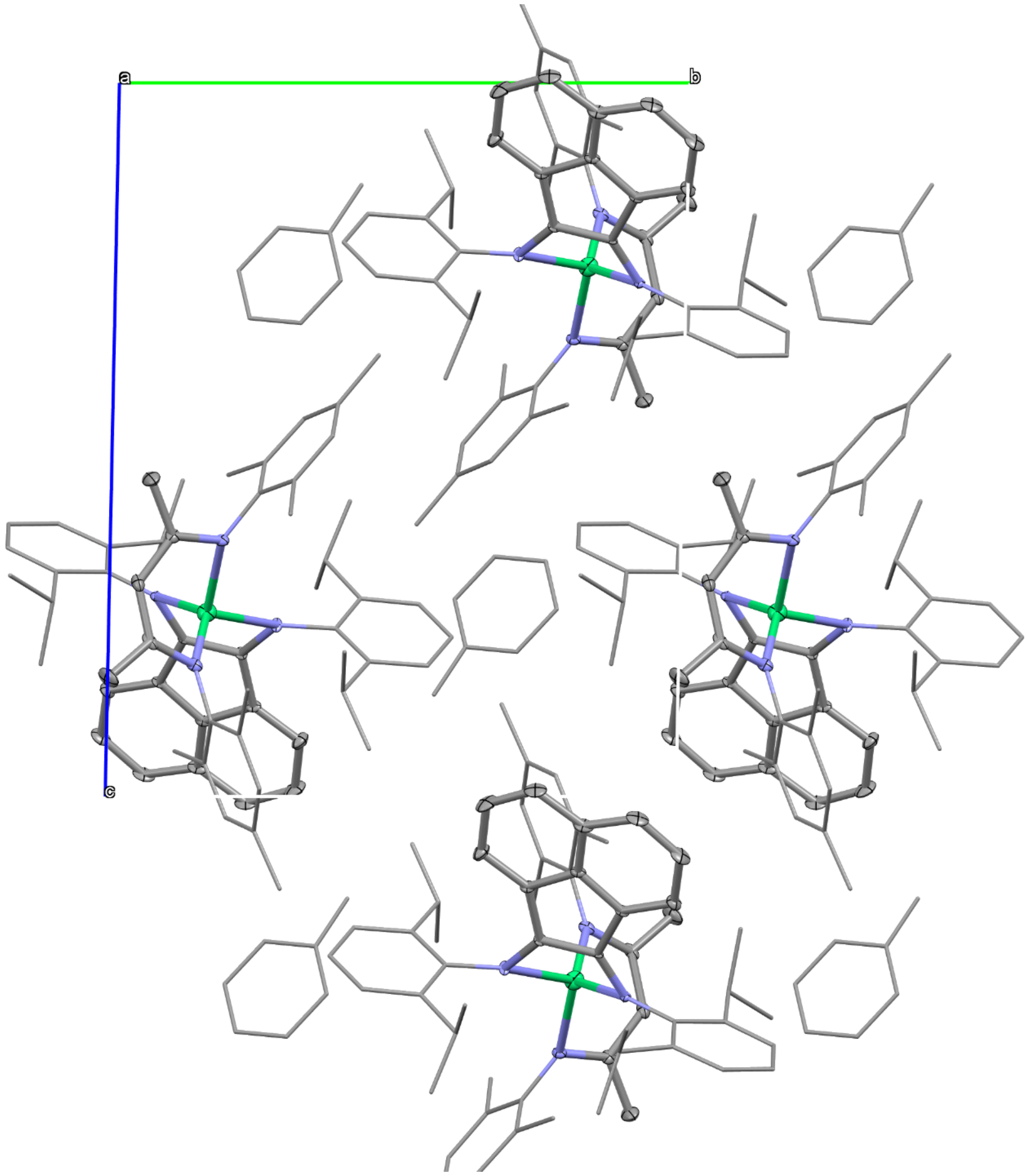

Figure S11. Crystal cell of (NacNac^{Mes})Er(BIAN^{dipp})·Toluene (**2**·Toluene) along a axis.

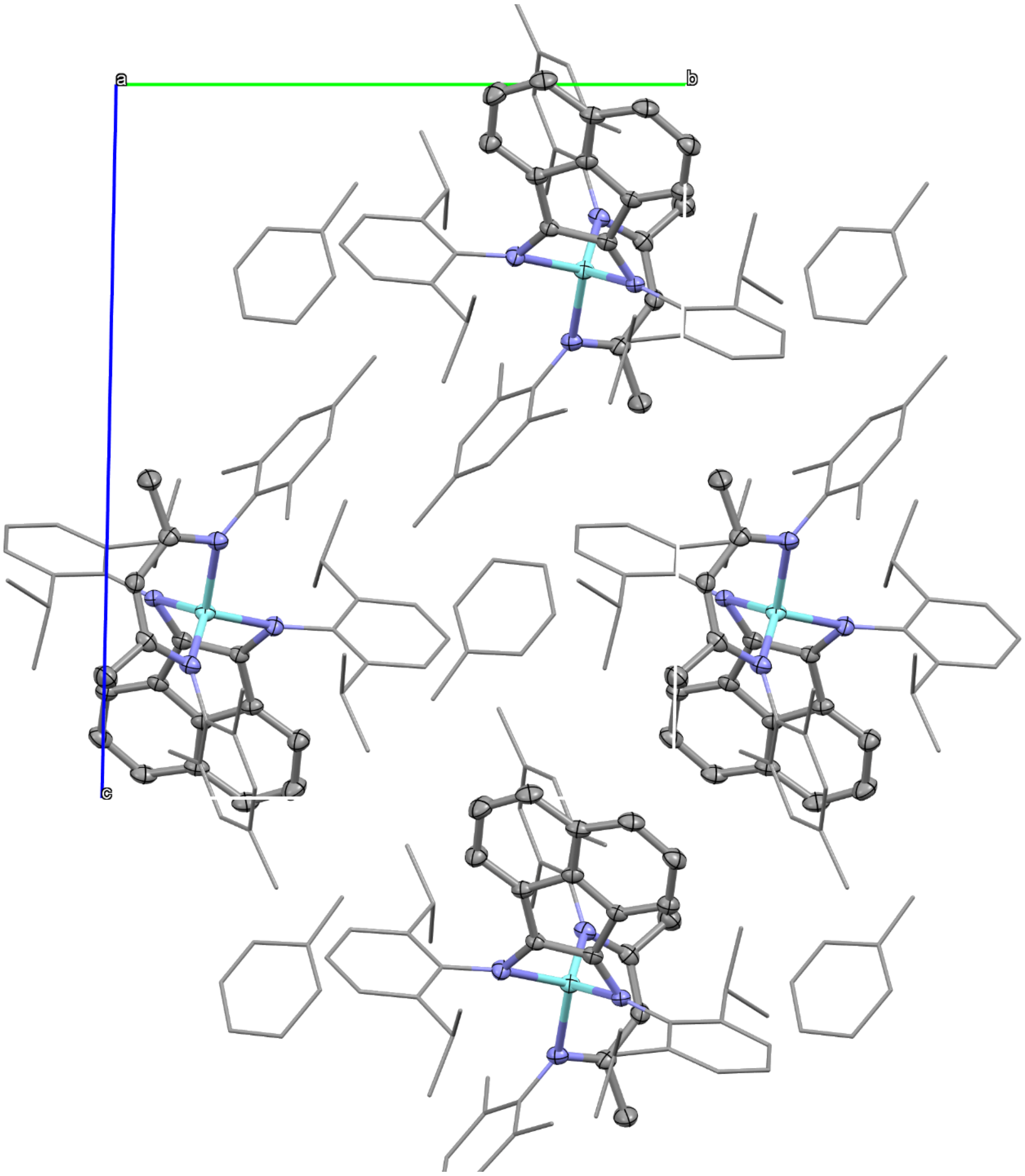

Figure S12. Crystal cell of (NacNac^{Mes})Y(BIAN^{dipp})·Toluene (**3**·Toluene) along a axis.

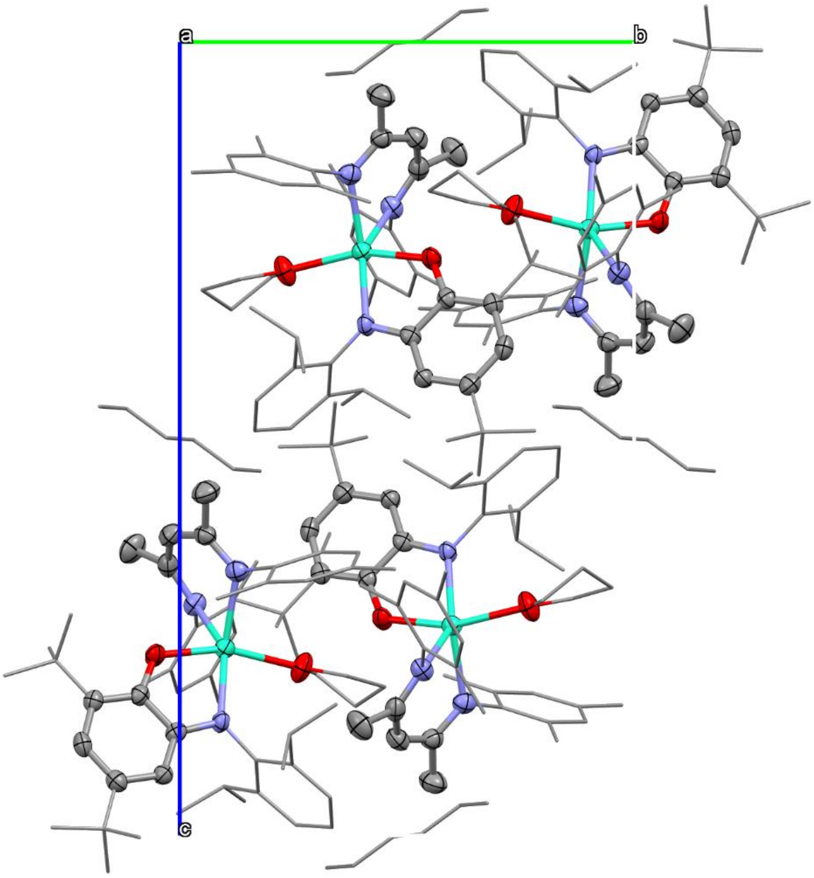

Figure S13. Crystal cell of (NacNac^{Mes})Dy(AP^{dipp})(THF)·0.5Hexane (**4**·0.5Hexane) along a axis.

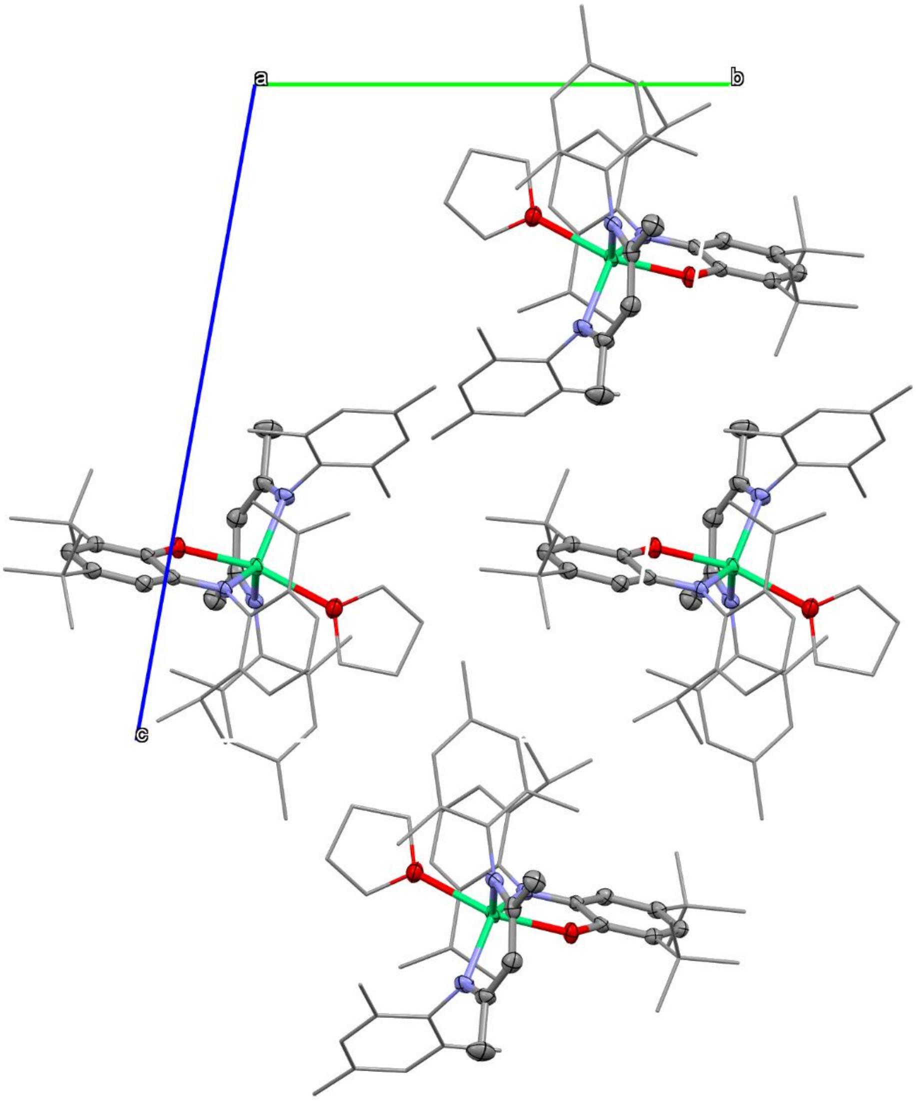

Figure S14. Crystal cell of (NacNac^{Mes})Er(AP^{dipp})(THF) (**5**) along a axis.

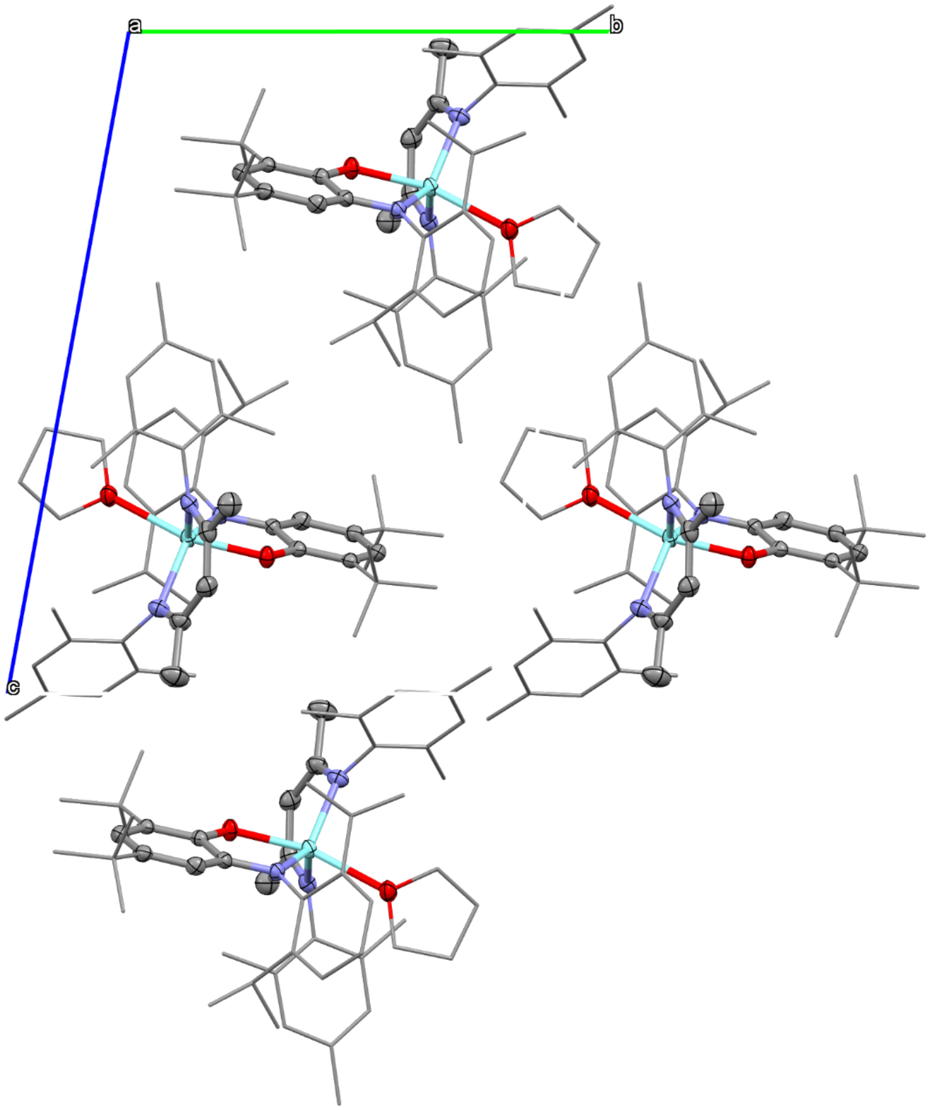

Figure S15. Crystal cell of (NacNac^{Mes})Y(AP^{dipp})(THF) (**6**) along a axis.

Table S1. Crystal data and structure refinement for the compounds.

Identification code	$(NacNac^{Mes})Dy(BIAN^{dipp})$	$(NacNac^{Mes})Er(BIAN^{dipp})\cdot$ Toluene	$(NacNac^{Mes})Y(BIAN^{dipp})\cdot$ Toluene	$(NacNac^{Mes})Dy(AP^{dipp})\cdot$ 0.5Hexane	$(NacNac^{Mes})Er(AP^{dipp})$	$(NacNac^{Mes})Y(AP^{dipp})$
	**1**	**2**·Toluene	**3**·Toluene	**4**·0.5Hexane	**5**	**6**
Empirical formula	$C_{59}H_{69}N_4Dy$	$C_{66}H_{77}ErN_4$	$C_{66}H_{77}N_4Y$	$C_{56}H_{81}DyN_3O_2$	$C_{53}H_{74}ErN_3O_2$	$C_{53}H_{74}N_3O_2Y$
Formula weight	996.68	1093.57	1015.22	990.73	952.41	874.06
Temperature / K	150(2)	150(2)	150(2)	200(2)	293(2)	150(2)
Space group	$P2_1/n$	P–1	P–1	$P2_1/c$	P–1	P–1
a / Å	12.0680(6)	12.2813(2)	12.2813(2)	19.552(4)	11.4962(7)	11.534(2)
b / Å	19.8718(10)	13.5420(2)	13.5420(2)	12.524(3)	12.7044(7)	12.740(3)
c / Å	20.9515(11)	17.3575(3)	17.3575(3)	22.503(5)	18.1034(10)	18.129(4)
α / °	90	90.1890(10)	90.1890(10)	90	95.834(5)	95.92(3)
β / °	93.8582(11)	103.1510(10)	103.1510(10)	103.49(3)	106.728(5)	106.92(3)
γ / °	90	94.0120(10)	94.0120(10)	90	102.926(5)	102.91(3)
Volume / $Å^3$	5013.1(4)	2803.64(8)	2803.64(8)	5358(2)	2428.6(3)	2443.4(10)
Z	4	2	2	4	2	2
ρ_{calc} / g·cm^{-3}	1.321	1.295	1.203	1.228	1.302	1.188
μ / mm^{-1}	1.532	1.540	1.083	1.435	1.769	1.234
F(000)	2068.0	1138.0	1080.0	2080.0	994.0	936.0
Crystal size / mm^3	0.12 × 0.1 × 0.1	0.4 × 0.1 × 0.1	0.4 × 0.15 × 0.15	0.18 × 0.15 × 0.15	0.2 × 0.15 × 0.12	0.14 × 0.12 × 0.1
Radiation	MoKα (λ = 0.71073)	MoKα (λ = 0.71073)	MoKα (λ = 0.71073)	MoKα (λ = 0.71073)	MoKα (λ = 0.71073)	MoKα (λ = 0.71073)
2Θ range / °	3.788 to 55.024	2.41 to 51.472	3.414 to 51.59	3.722 to 51.36	3.342 to 51.86	3.334 to 50.7
Index ranges	$-15 \le h \le 15$, $-25 \le k \le 25$, $-27 \le l \le 27$	$-14 \le h \le 14$, $-16 \le k \le 16$, $-21 \le l \le 21$	$-14 \le h \le 14$, $-16 \le k \le 13$, $-21 \le l \le 21$	$-23 \le h \le 23$, $-15 \le k \le 15$, $-27 \le l \le 27$	$-14 \le h \le 13$, $-15 \le k \le 14$, $-22 \le l \le 22$	$-13 \le h \le 13$, $-14 \le k \le 15$, $-21 \le l \le 19$
Reflections collected	44314	31457	25464	32333	22296	18596
Independent reflections	11509 [R_{int} = 0.0219, R_{sigma} = 0.0189]	10606 [R_{int} = 0.0346, R_{sigma} = 0.0387]	10533 [R_{int} = 0.0425, R_{sigma} = 0.0670]	10166 [R_{int} = 0.1093, R_{sigma} = 0.1030]	9055 [R_{int} = 0.0753, R_{sigma} = 0.0766]	8903 [R_{int} = 0.0700, R_{sigma} = 0.1064]
Data/restraints/parameters	11509/0/582	10606/12/628	10533/45/651	10166/42/598	9055/0/550	8903/0/550
Goodness-of-fit on F^2	1.046	1.073	1.033	0.883	0.946	0.884
Final R indexes [I>=2σ (I)]	R_1 = 0.0217, wR_2 = 0.0540	R_1 = 0.0435, wR_2 = 0.1238	R_1 = 0.0449, wR_2 = 0.1041	R_1 = 0.0416, wR_2 = 0.0866	R_1 = 0.0374, wR_2 = 0.0767	R_1 = 0.0459, wR_2 = 0.0836
Final R indexes [all data]	R_1 = 0.0263, wR_2 = 0.0553	R_1 = 0.0481, wR_2 = 0.1275	R_1 = 0.0685, wR_2 = 0.1128	R_1 = 0.0739, wR_2 = 0.0930	R_1 = 0.0459, wR_2 = 0.0784	R_1 = 0.0799, wR_2 = 0.0921
Largest diff. peak/hole / e·$Å^{-3}$	0.65/-0.37	1.69/-0.71	0.69/-0.50	0.81/-0.85	1.11/-1.60	0.58/-0.40

Table S2. Geometry analysis of the tetracoordinated lanthianide polyhedra in complexes **1–3** by SHAPE program*

	D_{4h} (Square)	T_d (Tetrahedron)	C_{2v} (Seesaw)	C_{3v} (Vacant trigonal bipyramid)
**1**	23.11	5.78	7.56	6.81
**2**·Toluene	24.87	6.73	7.65	8.44
**3**·Toluene	25.30	5.50	7.58	7.50

*SHAPE 2.1 program for the stereochemical analysis of molecular fragments by means of Continuous Shape Measures and associated tools. Llunell, M.; Casanova, D.; Girera, J.; Alemany, P.; Alvarez, S. SHAPE, version 2.1; Universitat de Barcelona: Barcelona, Spain, 2013. http://www.ee.ub.edu/

Table S3. Geometry analysis of the pentacoordinated lanthianide polyhedra in complexes **4–6** by SHAPE program*

	D_{5h} (Pentagon)	C_{4v} (Vacant octahedron)	D_{3h} (Trigonal bipyramid)	C_{4v} (Spherical square pyramid)	D_{3h} (Johnson trigonal bipyramid J12)
**4**·0.5Hexane	24.11	5.40	6.54	4.53	9.01
**5**	23.69	6.79	5.78	5.20	8.49
**6**	23.47	6.94	6.03	5.37	8.73

*SHAPE 2.1 program for the stereochemical analysis of molecular fragments by means of Continuous Shape Measures and associated tools. Llunell, M.; Casanova, D.; Girera, J.; Alemany, P.; Alvarez, S. SHAPE, version 2.1; Universitat de Barcelona: Barcelona, Spain, 2013. http://www.ee.ub.edu/

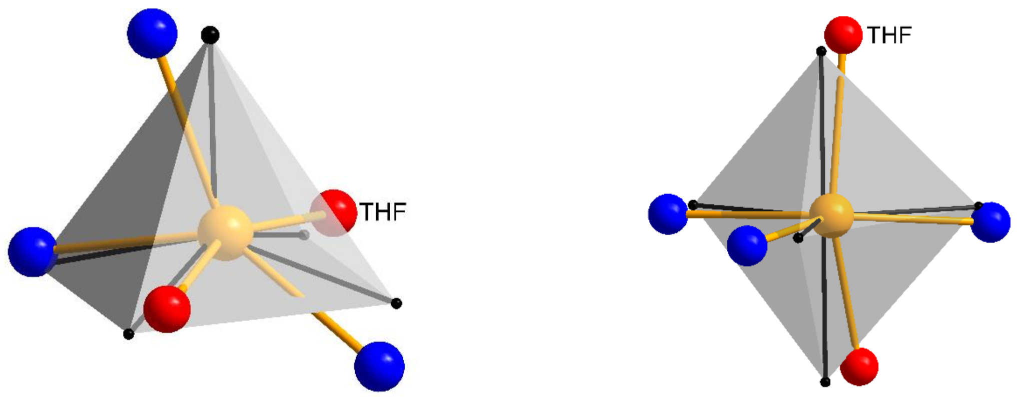

(a) (b)

Figure S16. Overlay of the coordination polyhedron in complexes **4–6** (by the example of **6**) and regular spherical square pyramid (a) and trigonal bipyramid (b) according to Continuous Shape Measures routine implemented in SHAPE program.
